# Survival enhancing indications for coronary artery bypass graft surgery in California

**DOI:** 10.1186/1472-6963-8-257

**Published:** 2008-12-16

**Authors:** Zhongmin Li, Richard L Kravitz, James P Marcin, Patrick S Romano, David M Rocke, Timothy A Denton, Ralph G Brindis, Jian Dai, Ezra A Amsterdam

**Affiliations:** 1 Division of General Internal Medicine and Center for Healthcare Policy and Research, University of California, Davis, Sacramento, CA, USA; 2 Department of Pediatrics, University of California, Davis, Sacramento, CA, USA; 3 Department of Biostatistics, University of California, Davis, Davis, CA, USA; 4 High Desert Heart Institute, Victorville, CA, USA; 5 Northern California Kaiser Permanente, Oakland, CA, USA; 6 Division of Cardiovascular Medicine, University of California, Davis, Sacramento, CA, USA

## Abstract

**Background:**

Coronary artery bypass graft (CABG) surgery is performed because of anticipated survival benefit, improvement in quality of life, or both. We performed this study to explore variations in clinical indications for CABG surgery among California hospitals and surgeons.

**Methods:**

Using California CABG Outcomes Reporting Program data, we classified all isolated CABG cases in 2003–2004 as having "probable survival enhancing indications (SEIs)", "possible SEIs" or "non-SEIs." Patient and hospital characteristics associated with SEIs were examined.

**Results:**

While 82.9% of CABG were performed for probable SEIs, the range extended from 68% to 96% among hospitals and 51% to 100% among surgeons. SEI rates were higher among patients aged ≥ 65 compared with those aged 18–64 (Adjusted Odds Ratio [AOR] > 1.29 for age groups 65–69, 70–74 and ≥ 75; all p < 0.001), among Asians and Native Americans compared with Caucasians (AOR 1.22 and 1.15, p < 0.001); and among patients with hypertension, peripheral vascular disease, diabetes, cerebrovascular disease and congestive heart failure compared to patients without these conditions (AOR > 1.09, all p < 0.001). Variations in indications for surgery were more strongly related to patient mix than to surgeon or hospital effects (intraclass correlation [ICC] = 0.04 for hospital; ICC = 0.01 for surgeon).

**Conclusion:**

California hospitals and surgeons vary in their distribution of indications for CABG surgery. Further research is required to identify the roles of market factors, referral patterns, patient preferences, and local clinical culture in producing the observed variations.

## Background

Randomized controlled trials and prospective cohort studies have shown that coronary artery bypass graft (CABG) surgery is associated with increased survival in selected patients [1-3]. Nonetheless, the procedure entails substantial cost [4] and a risk of perioperative mortality ranging from 2% to 5% [5-11]. Therefore, careful patient selection is necessary to ensure that this procedure is performed on patients for whom the benefits (including, but not limited to mortality reduction) are likely to exceed the risks. In general, randomized trials favor surgery over medical therapy or percutaneous coronary intervention (PCI) for patients with left main or multivessel coronary artery disease (CAD) with extensive jeopardized myocardium. For other patients, evidence for survival benefit is less certain, and quality of life considerations dominate [12-21].

CABG surgery is performed because of anticipated survival benefit, improvement in quality of life, or both. The focus of this study is on the subset of CABG surgeries that are performed because of anticipated survival benefit. At the level of an individual hospital or surgeon, either a very low or very high proportion of CABG surgeries performed for survival enhancing indications (SEIs) could suggest the need for further inquiry. For example, a low SEI proportion could result from operating on an excess of patients with equivocal indications for surgery or on too few patients with SEIs. An extremely high SEI proportion, in contrast, could mean that the hospital or surgeon is disinclined or unable to offer surgery to patients who might benefit from CABG surgery in terms of quality of life, or that an aggressive percutaneous intervention program is diverting most of these patients.

We performed this study to examine variation in patient selection for CABG surgery among California hospitals and surgeons. Specifically, we tested the following hypotheses: 1) the proportion of CABG performed for survival-enhancing indications varies across hospitals, across surgeons, and across metropolitan areas; 2) given known gender and racial/ethnic disparities in overall rates of CABG surgery, a higher proportion of CABG is performed for SEIs (versus more discretionary indications) among women than among men, among non-Caucasian than among Caucasian patients, and among patients with more comorbidities such as hypertension, diabetes and congestive heart failure; and 3) given that PCI and CABG are acceptable options for most patients without SEIs, a higher proportion of CABG is performed for SEIs (versus more discretionary indications) at hospitals with high PCI volume (or PCI/CABG volume ratio) than at hospitals with low PCI volume, and at teaching hospitals than at non-teaching hospitals.

## Methods

Data were obtained from the California CABG Outcomes Reporting Program (CCORP), managed by California Office of Statewide Health Planning and Development [9], which requires California hospitals to submit detailed clinical information on indications and outcomes for CABG surgery under the State mandate (SB680, 2001). During 2003 and 2004, 121 hospitals submitted data including patient demographics, clinical characteristics, and observed in-hospital mortality. Hospital data were linked to the state vital statistics file from California Department of Health Services (DHS) to identify patients who died outside the operating hospital within the 30 days following CABG surgery. The data collection system, based on specifications from the Society of Thoracic Surgeons, includes a multi-step cleaning process and annual onsite audit to ensure data accuracy. Isolated CABG surgeries, defined as CABG surgery performed without other major cardiac procedures such as valve repair or replacement during the same operation, were selected for public reporting of risk-adjusted operative mortality by hospital and surgeon. The CCORP data collection procedures and analysis methodology are described in detail elsewhere [9].

### Classification of Indications for CABG Surgery

The CCORP data collection system includes questions on the presence or absence of left main CAD, number of diseased coronary vessels, left ventricular ejection fraction (LVEF) and Canadian Cardiovascular Society classification of angina severity. Hospitals are not required to report whether the proximal left anterior descending (PLAD) artery was stenotic or bypassed. Accordingly, we classified all isolated CABG surgeries performed in 2003 and 2004 into one of three indication categories, based on American College of Cardiology (ACC)/American Heart Association (AHA) clinical guidelines [12,13]: 1) "probable survival enhancing indications (SEIs)"; 2) "possible SEIs" and 3) "non SEIs" (ie., "quality of life indications" only). Probable SEIs included patients with: 1) left main CAD (stenosis > 50%) and 2) 3-vessel CAD. Possible SEIs included patients with 2-vessel CAD with diminished LVEF (< 50%) or 2-vessel CAD with normal ejection fraction (≥ 50%) but with severe angina (Class 3/4) (assuming PLAD involvement). Non-SEIs included patients with 2-vessel disease with normal LVEF (≥ 50%) and no/mild angina (Class 0/1/2) (assuming no PLAD involvement) or with 1-vessel or unknown disease (Table 1).

**Table 1:** Survival enhancing indications (SEIs) for coronary artery bypass surgeries in California, 2003–2004

**Patient Characteristics**	**N**	**%**	**SEI Status**
Left main coronary artery disease (CAD) (Stenosis > 50%)	10,102	25.0	Probable SEIs (N = 33,494, 82.9%)
3-vessel CAD	23,392	57.9	

2-vessel CAD with a diminished LVEF (< 50%)	1,458	3.6	Possible SEIs (N = 3,985, 9.9%)
2-vessel CAD with normal LVEF (=> 50%) but Angina class 3/4	2,527	6.3	

2-vessel CAD with normal LVEF (=> 50%), Angina class 0/1/2	1,441	3.6	Non-SEIs (N = 2,895, 7.2%)
1-vessel CAD	1,402	3.5	
Missing value	52	0.1	

Total	40,374	100.0	

CAD: coronary artery disease; LVEF: left ventricular ejection fraction;

SEIs: survival enhancing indications

### Statistical Analysis

For descriptive purposes we examined the proportion of CABG operations performed for probable SEIs, possible SEIs and non-SEIs by hospital and by surgeon (restricted to 225 surgeons performing at least 50 procedures per year, which we chose as an arbitrary threshold reflecting an "active" surgical practice). To avoid the complexity of multinomial logistic regression, we dichotomized indications for each CABG surgery in two alternative ways: 1) probable SEIs versus possible and non-SEIs (shown in the tables below), and 2) probable and possible SEIs versus non-SEIs. The second approach assumes that all patients with 2-vessel disease and diminished LVEF or severe angina had PLAD involvement whereas the first approach assumes that none did. Neither of these assumptions is completely correct, but they place bounds on the range containing the true value. Our results from these two approaches were quite similar (e.g., the Spearman rank correlation at the hospital level was 0.82, 95% confidence interval (CI): 75.3%–87.2%), so we present the results of the first approach only.

We used the standard logistic regression model to examine whether age, gender, race/ethnicity and preoperative clinical characteristics were associated with the likelihood of undergoing CABG surgery for a SEI. Furthermore, we estimated hierarchical logsitic regression models and used the results to evaluate the variation attributable to the patient and to the healthcare provider. The standard logistic regression model is

$$\begin{array}{l} Y_{ij}=p_{ij}+e_{ij},\\ logit(p_{ij})=\log\left(\frac{p_{ij}}{1-p_{ij}}\right)=\mu +\sum_{k=1}^{K}\beta_{k}X_{k,ij} \end{array}$$

where Y is a binary outcome variable for patient survival enhancing indication and follows the Bernoulli distribution, *Y*~*Bin*(1, *π*); X's are patient-level predictors; *μ *and *β's *are the regression coefficients; *i *= 1, ..., *I*_*j *_is the patient level indicator; *j *= 1, ..., *J *is the hospital level indicator; and *p*_*ij *_is the probability of survival enhancing indication for patient i in hospital j, conditional on the risk factors X's. The model assumes that patient level random errors *e*_*ij *_are independent with moments *E*(*e*_*ij*_) = 0 and $Var(e_{ij})=\sigma_{e}^{2}=p_{ij}(1-p_{ij})$. The two-level random intercept model is an extension of the standard logistic model and treats the hospital intercepts as a random variable in the linear function. In the random hospital intercept model, the logit is:

$$logit(p_{ij})=\mu +\sum_{k=1}^{K}\beta_{k}X_{k,ij}+u_{j}$$

where *u*_*j *_are the hospital random effects and it is assumed that $u_{j}\sim iid\;N(0,\sigma_{u}^{2})$. It is straightforward to extend the 2-level model to 3-level models that include both hospital-level and surgeon-level random effects. The models used in this paper were fitted with SAS GLIMMIX.

Based on previous studies of variation in overall CABG surgery utilization, we hypothesized that the SEI proportion would be higher among older patients than among younger patients, higher among women than among men, higher among minority patients than among Caucasian patients, and higher among patients with more preoperative comorbidities. We further hypothesized that teaching hospitals and other hospitals with active PCI programs (e.g., hospitals with high PCI volume or a high ratio of PCI to CABG volume) would have high SEI proportions, because most patients with only quality-of-life indications would be referred for PCI instead of CABG. Hospital regions (San Francisco Bay area, Greater Los Angeles area, Greater San Diego area, and other regions) were entered into the model as "nuisance" variables to adjust for regional differences in the relative availability of cardiologists and thoracic surgeons.

We considered three sources of variation in the hierarchical logistic model for SEIs in CABG surgery: variation attributable to the patient, the surgeon and the hospital. Specifically, we calculated an intraclass correlation (ICC) coefficient, which describes the fraction of residual variance (unexplained variation) from the regression on patient characteristics that is accounted for by differences among hospitals and/or surgeons [22,23]. For the two-level logistic model, we estimated the approximate ICC as follows:

$$ICC\approx \frac{\sigma_{u}^{2}}{\sigma_{u}^{2}+\pi^{2}/3}$$

where $\sigma_{u}^{2}$ is the second level estimated variance and *π *is the quantity 3.14159. This method is simple to use but can produce different estimates of the ICC than other methods [24,25]. For the logistic models with three levels, we used an analogous method. Denoting the estimated variances of hospitals and surgeons as $\sigma_{h}^{2}$ and $\sigma_{s}^{2}$, respectively, the ICC values for hospital (h) and surgeon (s) were calculated as:

$$\begin{array}{ccc} ICC_{h}\approx \frac{\sigma_{h}^{2}}{\sigma_{h}^{2}+\sigma_{s}^{2}+\pi^{2}/3} & \text{and} & ICC_{s}\approx \frac{\sigma_{s}^{2}}{\sigma_{h}^{2}+\sigma_{s}^{2}+\pi^{2}/3} \end{array}$$

The hierarchical model used for computing ICC does not contain hospital or surgeon level variables. In a separate model, we calculated hospital random effects to identify hospitals that were either significantly higher or lower in SEI proportion when compared to the statewide average, controlling for patient characteristics. All data analyses were conducted using SAS version 9.1.3 (Cary, NC). The authors had full access to the data and take responsibility for its integrity.

## Results

During 2003 and 2004 study period, 302 surgeons performed 40,374 isolated CABG procedures in 121 California hospitals. Overall, 82.9% of CABG operations were performed for probable SEIs, 9.9% for possible SEIs and 7.2% for non-SEIs. Fifty-two cases could not be classified due to missing values. Excluding 4,739 CABG patients with emergent status or prior PCI procedure(s), 83.1% of CABG operations were performed for probable SEIs, 9.7% for possible SEIs and 7.2% for non-SEIs. Over two-thirds of probable SEI cases were comprised of patients with 3-vessel CAD, while the remainder had left main disease (Table 1). Non-SEI cases were equally divided between patients with single vessel disease and patients with two-vessel disease who had normal left ventricular function and mild or no angina.

**Figure 1:**
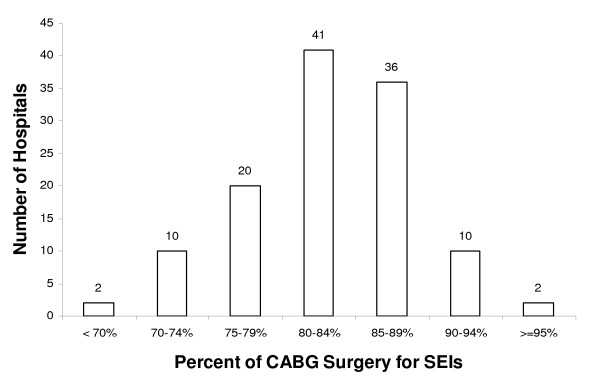
**Distribution of hospitals by CABG surgery for probable survival enhancing indications, California, 2003–2004**. SEIs = survival enhancing indications, CABG = Coronary Artery Bypass Graft.

**Figure 2:**
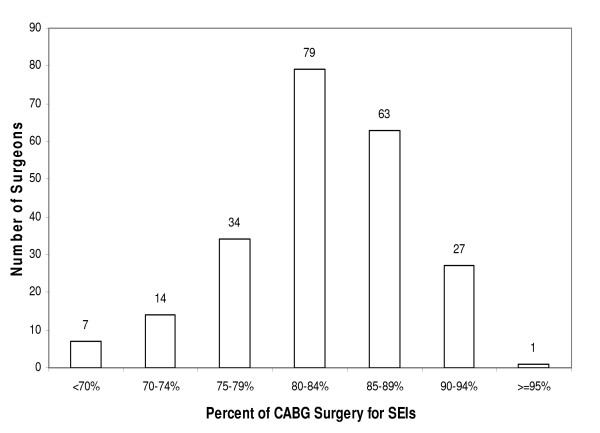
**Distribution of surgeons by CABG surgery for probable survival enhancing indications, California, 2003–2004**. (Note: Limited to 225 surgeons performing at least 50 CABG surgeries a year). SEIs = survival enhancing indications, CABG = Coronary Artery Bypass Graft.

There was substantial variation in the proportion of CABG surgeries performed for probable SEIs by hospital (mean 83% of CABG operations for a SEI, range 68–96%). The "probable SEI" rate exceeded 90% in 12 hospitals, whereas in 12 others the SEI rate was less than 75% (Figure 1). Variation among surgeons was also substantial (mean 84%, range 51–100%). Among the 225 surgeons performing at least 50 CABG surgeries a year, 28 had "probable SEI" rates exceeding 90% and 21 had rates below 75% (Figure 2). Using a hierarchical model with hospital random effects, 18 of 121 hospitals had "probable SEI" proportions significantly lower than the statewide average, adjusting for patient demographic and preoperative clinical characteristics (adjusted odds ratio [AOR] < 1, p < 0.05), and 17 hospitals had significantly higher "probable SEI" proportions (AOR > 1, p < 0.05) (data not shown).

Using hierarchical logistic regression in which patient demographic and clinical characteristics served as level I variables and hospital characteristics as level II variables, the likelihood of undergoing CABG surgery for a probable SEI was increased among patients in older age groups compared with patients aged 18–64 (for age 65–69: AOR = 1.42, 95% CI 1.32–1.52; for 70–74: AOR = 1.29, 95%CI 1.20–1.40; and for age ≥ 75: AOR = 1.29, 95%CI 1.19–1.39; all p < 0.001) (Additional file 1). Probable SEI was less likely among women than among men (AOR = 0.67, 95% CI 0.63–0.71, p < 0.001) but more likely among Asians and Native Americans than among Caucasians (AOR for Asian = 1.22, 95% CI 1.10–1.35, p < 0.001; AOR for Native American = 1.15, 95% CI 1.01–1.30, p < 0.05). Patients with hypertension, peripheral vascular disease (PVD), diabetes, cerebrovascular disease (CVD) or congestive heart failure (CHF) were more likely to have CABG for probable SEI (AOR for hypertension = 1.09, 95% CI 1.02–1.16; AOR for pvd = 1.32, 95% CI 1.21–1.44; AOR for diabetes = 1.25, 95% CI 1.18–1.33; AOR for CVD = 1.23, 95% CI 1.13–1.35; and AOR for CHF = 1.30, 95% CI 1.20–1.40; all p < 0.01). Controlling for patient demographic and clinical characteristics, hospital characteristics (including hospital teaching status, geographic region, CABG volume, PCI volume, PCI/CABG volume ratio in 2003–2004 and number of performing surgeons and mean surgeon volume within hospital) were not associated with the likelihood of having CABG surgery for probable SEIs. The hospital intraclass correlation coefficient, while significant, was of relatively small magnitude (ICC = 0.044, 95%CI = 0.030–0.058, p < 0.001), as was the surgeon intraclass correlation (ICC = 0.01, 95%CI = 0.003–0.013, p < 0.001).

To explore differences in patient selection that might account for the observed between-hospital variation in SEI rates, we compared aggregated patient characteristics by hospital group (Additional file 2). Compared with hospitals in the lowest 25th percentile for SEI rate, hospitals in the upper 25th percentile cared for patients who were younger and less likely to undergo CABG for emergent indications but more likely to be non-Caucasian, to have had a recent myocardial infarction, to have an ejection fraction < 40%, and to have left main or 3-vessel CAD (Table 3).

## Discussion

The results of this study show that the vast majority of the 40,374 isolated CABG surgeries performed during 2003 and 2004 in California were associated with "probable" or "possible" survival enhancing indications. The findings are consistent with data obtained from more detailed clinical studies of the clinical appropriateness of CABG surgery [13-15], and are, in the aggregate, reassuring. However, hospitals varied significantly in terms of the severity of CAD selected for bypass surgery. Most CABG operations were for SEIs, but in 32 hospitals (26%), patients lacking a probable SEI constituted at least one-fifth of patients taken to surgery.

We used a hierarchical logistic regression model on hospital characteristics and patient demographics to estimate ICC values, which measures the fraction of variation (residual variance) that is explained by hospital or surgeon or both. We found that these fractions were small for hospital and surgeon, suggesting that patient mix might be more important than hospital and surgeon effects in explaining the variation in use of CABG surgery for SEI. We also found that the ICC value for hospitals was larger than the value for surgeons, suggesting that some elements of cardiac surgical decision making might be related more directly to hospital referral patterns and culture than to the judgment of individual surgeons.

For a number of reasons, the proportion of surgeries performed for "probable SEIs" is not a strong measure of quality or appropriateness at the level of the individual surgeon or hospital, but this metric may still be useful as a window into the critical issue of patient selection. As a ratio measure, the SEI proportion simultaneously reflects the tendency of providers to perform CABG surgery on patients with SEIs and to eschew operation on patients lacking SEIs. Thus, a low SEI rate could indicate failure to operate on eligible patients with extensive CAD, enthusiastic acceptance of patients with minimal disease, or both. In addition, hospitals and their surgeons may vary in terms of local referral patterns. An aggressive PCI program, for example, could siphon away all but the 3-vessel and left main disease patients, resulting in a high SEI proportion, whereas in areas with fewer interventional cardiologists, patients may be preferentially shunted towards surgery, resulting in a low SEI proportion. Our prior beliefs as to the direction of this effect were not strong; in fact, it could be argued that a hospital with an enthusiastic PCI program would have a low SEI proportion if interventionists were handling patients with 3-vessel and left main coronary disease in preference to CABG surgery. Despite these limitations, it is reasonable to hypothesize that hospitals with a high SEI rate have a low incidence of inappropriate surgery; no conclusions can be drawn about hospitals with low SEI rates. In any case, the most important application of the SEI metric will be to stimulate individual hospitals to examine their own data in support of internal quality improvement.

During the 1980s and 1990s, RAND developed appropriateness criteria [26] for CABG surgery. In a subsequent multi-institutional study, 74–95% of CABG surgeries were deemed necessary or crucial [27-29]. Our results are consistent with the RAND findings. However, the CCORP (and STS) data currently lack certain information available to RAND investigators, including left anterior descending (LAD) coronary artery involvement and the intensity of medical management. As a result of this study, the Clinical Advisory Panel, the oversight body of the CCORP, has approved recommendations to add LAD involvement as a required new element for statewide data collection.

In the multivariable analysis, patients in older age groups (over age 65) undergoing CABG surgery were more likely than younger patients to have a "probable SEI." Similarly, CABG patients with significant chronic comorbidities such as hypertension, PVD, diabetes, CVD and CHF were more likely than CABG patients without those comorbidities to have a "probably SEI." The most plausible explanation for this finding is that surgeons may "screen out" elderly and chronically ill patients without SEIs [due to concern about their perioperative risk in the setting of uncertain survival benefit) and refer them back to cardiologists for possible PCI or medical therapy. Of course, primary care physicians and cardiologists may also be less likely to refer elderly and chronically ill patients without SEIs to surgeons, for the same reason. The higher "probable SEI" rate among men than women was unexpected, but may reflect unmeasured gender differences in the spectrum of coronary disease (e.g., prevalence of LAD involvement with one or two-vessel disease).

In summary, the "probable SEI" proportion varies substantially among California hospitals that perform CABG surgery. Extreme values of this metric do not necessarily indicate a problem with quality. In particular, we need to know whether low SEI rates at the hospital level correlate with clinical inappropriateness, using more detailed methods such as the RAND approach. Additional research is also needed to determine whether the observed variation in patient selection results from market factors, referral patterns, patient preferences, or local clinical culture. Also, hospitals with SEI proportions at the extreme high- or low-end of the distribution may wish to examine their own data in more detail to assure themselves that patient selection is occuring in accord with current standards of evidence and practice.

## Competing interests

The authors declare that they have no competing interests.

## Authors' contributions

ZL, RK and PR participated in the study design, data analysis and drafted the manuscript. EA, JM, TD and RB provided clinical advice and participated in the munuscript editing. JD and DR participated in development of statistical models.

## Pre-publication history

The pre-publication history for this paper can be accessed here:



## Supplementary Material

Additional file 1 **Table 2. **Hierarchical logistic regression on isolated CABG surgery survival enhancing indication, California, 2003–2004. Click here for file

Additional file 2 **Table 3**. Comparison of patient clinical profile among hospital SEI groups. Click here for file
