# Supplementary material for: Survival enhancing indications for coronary artery bypass graft surgery in California
Source: BMC Health Serv Res. 2008 Dec 16;8:257. doi: 10.1186/1472-6963-8-257 (PMC2621199; doi:10.1186/1472-6963-8-257)
Supplement: Additional file 1 — Table 2. Hierarchical logistic regression on isolated CABG surgery survival enhancing indication, California, 2003–2004. [file 1472-6963-8-257-S1.doc]

| Table 2: Hierarchical Logistic Regression on Isolated CABG Surgery Survival Enhancing Indication, California, 2003-2004 | | | | | | |
| --- | --- | --- | --- | --- | --- | --- |
| Variable | | Prevalence | Adjusted Odds Ratio( AOR) | AOR: 95% Confidence Limits | | p-value |
| Hospital Characteristics (N=121) | |  |  |  |  |  |
|  | Teaching (vs. Non-teaching) | 14.9% | 1.114 | 0.879 | 1.412 | 0.371 |
|  | Isolated CAB Volume (per 10 additional procedures), Median | 260 | 0.998 | 0.991 | 1.005 | 0.518 |
|  | PCI volume (per 20 additional procedures), Median | 405 | 1.000 | 0.993 | 1.008 | 0.923 |
|  | PCI/CAB Volume Ratio, Mean | 1.8 | 1.063 | 0.951 | 1.187 | 0.284 |
|  | San Francisco Bay Area and San Jose | 19.8% | 1.045 | 0.983 | 1.111 | 0.416 |
|  | Greater Los Angeles Area | 31.4% | 1.014 | 0.976 | 1.053 | 0.747 |
|  | Greater San Diego | 9.1% | 0.908 | 0.720 | 1.145 | 0.412 |
|  | Other Region | 39.7% | Reference |  |  |  |
| Surgeon Characteristics (N=302) | |  |  |  |  |  |
|  | # of Surgeons per Hospital, Mean | 5.0 | 0.967 | 0.786 | 1.188 | 0.161 |
|  | Mean Surgeon Volume within Hospital (per 10 additional procedures) | 73.5 | 1.142 | 0.831 | 1.569 | 0.486 |
| Patient Characteristics (N=40,374) | |  |  |  |  |  |
| Age | => 75 | 25% | 1.287 | 1.191 | 1.392 | <.0001 |
|  | 70-74 | 16.2% | 1.293 | 1.195 | 1.399 | <.0001 |
|  | 65-69 | 16.5% | 1.415 | 1.318 | 1.518 | <.0001 |
|  | 18-64 | 42.3% | Reference |  |  |  |
| Gender | Female (vs. Male) | 26.5% | 0.666 | 0.628 | 0.707 | <.0001 |
| Race/Ethnicity | Asian | 8.8% | 1.220 | 1.099 | 1.354 | 0.001 |
|  | African American | 3.7% | 1.041 | 0.900 | 1.204 | 0.590 |
|  | Hispanic | 12.1% | 1.087 | 0.995 | 1.188 | 0.064 |
|  | Native American | 5.4% | 1.149 | 1.012 | 1.304 | 0.032 |
|  | Caucasian | 70.0% | Reference |  |  |  |
| *Hypertension* |  | 78.8% | 1.090 | 1.022 | 1.163 | 0.009 |
| *Dialysis* |  | 2.6% | 1.034 | 0.865 | 1.235 | 0.717 |
| Peripheral Vascular Disease | | 13.7% | 1.316 | 1.206 | 1.437 | <.0001 |
| Cerebrovascular Disease | | 12.8% | 1.233 | 1.128 | 1.347 | <.0001 |
| *Diabetes* |  | 38.8% | 1.254 | 1.183 | 1.329 | <.0001 |
| *Severe Chronic Lung Disease* | | 2.8% | 1.107 | 0.926 | 1.324 | 0.263 |
| *Congestive Heart Failure* | | 17.7% | 1.297 | 1.200 | 1.402 | <.0001 |
| Note: Without hospital or surgeon level variables, the hospital and surgeon random effect model has Intraclass correlation (ICC) for hospital: ICC=0.044, 95%CI=0.030-0.058, p<0.001; for surgeon ICC=0.008, 95%CI=0.003-0.013, p<0.001. | | | | | | |

CABG: coronary artery bypass graft surgery; ICC: Intraclass correlation; PCI: percutaneous coronary intervention; SEIs: survival enhancing indications.
