# Supplementary material for: Survival enhancing indications for coronary artery bypass graft surgery in California
Source: BMC Health Serv Res. 2008 Dec 16;8:257. doi: 10.1186/1472-6963-8-257 (PMC2621199; doi:10.1186/1472-6963-8-257)
Supplement: Additional file 2 — Table 3. Comparison of patient clinical profile among hospital SEI groups. [file 1472-6963-8-257-S2.doc]

| Table 3: Comparison of Patient Clinical Profile Among Hospital SEI Groups | | | | | |
| --- | --- | --- | --- | --- | --- |
|  |  | Hospital Group per SEI Rate | | |  |
|  |  | Lower 25 Percentile | 25-75 percentile | Upper 25 percentile | Overall P-value |
| Hospital, N |  | 31 | 60 | 30 |  |
| Patient, N |  | 10,912 | 20,930 | 8,532 |  |
| Age, mean (SD§) | | 66.4 (10.7) | 66.1 (10.7) | 66.0 (10.8)* | 0.044 |
| Female, % (SD) | | 26.8 (44.3) | 26.3 (44.0) | 26.7 (44.2) | 0.536 |
| Non-Caucasian, % (SD) | | 27.8 (44.8) | 29.7 (45.7)‡ | 33.7 (47.3)‡ | <.0001 |
| Acuity: Emergent/Salvage, % (SD) | | 6.1 (23.9) | 5.9 (23.5) | 4.7 (21.1)‡ | <.0001 |
| Hypertension, % (SD) | | 78.8 (40.9) | 78.6 (41.0) | 79.2 (40.6) | 0.610 |
| Dialysis, % (SD) | | 2.6 (16.0) | 2.7 (16.2) | 2.5 (15.5) | 0.537 |
| Peripheral Vascular Disease, % (SD) | | 13.9 (34.6) | 13.3 (34.0) | 14.3 (35.0) | 0.056 |
| Cerebrovascular Disease, % (SD) | | 12.8 (33.4) | 12.6 (33.2) | 13.3 (34.0) | 0.192 |
| Diabetes, % (SD) | | 39.1 (48.8) | 38.9 (48.7) | 38.2 (48.6) | 0.405 |
| Chronic Lung Disease: Severe, % (SD) | | 2.0 (14.1) | 3.1 (17.3)‡ | 3.0 (17.1)‡ | <.0001 |
| Myocardial Infarction, % (SD) | ≥21 days ago | 18.0 (38.4) | 17.4 (37.9) | 18.6 (38.9) | 0.050 |
|  | 8-20 days ago | 3.5 (18.5) | 4.4 (20.6)‡ | 4.3 (20.3)‡ | 0.001 |
|  | 1-7 days ago | 21.7 (41.2) | 21.9 (41.3) | 21.1 (40.8) | 0.370 |
|  | Within 24 Hours | 4.5 (20.8) | 4.7 (21.1) | 4.7 (21.1) | 0.819 |
| Congestive Heart Failure, % (SD) | | 15.6 (36.3) | 18.3 (38.7)‡ | 18.9 (39.1)‡ | <.0001 |
| Ejection Fraction <40%, % (SD) | | 16.7 (37.3) | 18.8 (39.1)‡ | 20.3 (40.2)‡ | <.0001 |
| Left Main Disease (>50% Stenosis), % (SD) | | 22.8 (41.9) | 24.6 (43.1) | 28.9 (45.3)‡ | <.0001 |
| Number of Diseased Vessels: ≥3, % (SD) | | 70.7 (45.5) | 78.8 (40.9)† | 86.4 (34.3)‡ | <.0001 |
| Predicted Mortality, % (SD) | | 3.05 (5.16) | 3.09 (5.48) | 3.10 (5.40) | 0.743 |
| Probable SEI rate, % (SD) | | 76.1(42.7) | 83.6 (37.0)‡ | 90.1 (29.8)%‡ | <.0001 |
| * Significant at p<0.05; † significant at P<0.01; ‡ significant at p<0.001. Hospital group of lower 25 percentile in SEI rate is the reference category for all multiple comparisons.  § Standard Deviation. | | | | | |
